# Structural and Functional Modifications of Hazelnut Proteins Induced by Atmospheric Cold Plasma

**DOI:** 10.3390/polym18030413

**Published:** 2026-02-05

**Authors:** Suzan Uzun

**Affiliations:** Food Engineering Department, Agricultural Faculty, Tekirdag Namik Kemal University, Tekirdag 59030, Türkiye; suzanuzun@nku.edu.tr

**Keywords:** atmospheric cold plasma, hazelnut protein, protein modification, plant proteins, physicochemical properties, techno-functional properties

## Abstract

This study evaluated the effects of atmospheric cold plasma (ACP) treatment duration on the physicochemical and functional properties of hazelnut protein. Proteins were extracted from defatted hazelnut flour and subjected to ACP for 0, 2, 4, 6, and 8 min. The results demonstrated that ACP treatment significantly modified protein characteristics: it generally reduced particle size and increased absolute zeta potential, with the smallest particles observed after 4 and 6 min of treatment. Concurrently, a decrease in L, a, and b color values indicated sample darkening with extended processing. Structural analysis revealed that ACP induced changes in protein secondary structure, leading to a significant increase in surface hydrophobicity and a decrease in free sulfhydryl content. These structural and physicochemical modifications, particularly the enhanced surface hydrophobicity and reduced particle size, collectively improved emulsifying activity and stability, as well as foaming capacity and stability. The highest emulsion and foaming stability were observed in samples treated for 6 min. Hazelnut protein gels exhibited pronounced solid-like behavior and ACP treatment enhanced the rheological properties of the gels, with the maximum gel strength observed at a 6 min treatment. Overall, these findings indicate that ACP is an effective non-thermal technology for positively altering the physicochemical and techno-functional properties of hazelnut protein.

## 1. Introduction

Proteins are vital dietary macromolecules essential for human health, and the projected growth of the global population to nearly 10 billion by 2050 [1] is driving a rapidly growing demand for them. Meeting this demand through large-scale production presents a significant challenge. Furthermore, a growing number of consumers are rejecting animal proteins due to specific dietary preferences, religious practices, or ethical considerations [2]. In this context, the search for healthier and more sustainable protein sources has become a central focus in food science and agriculture. Plant-based proteins are increasingly considered the primary alternative to meet this demand, owing to their wide availability, lower production costs, and inherent alignment with diverse consumer principles. The economic potential of this shift is substantial, with the global plant-based food market projected to reach $162 billion by 2030 [3]. Therefore, the systematic exploration and development of novel plant protein resources is not only a promising avenue for fulfilling human nutritional requirements but also a critical step toward building a more sustainable and economically resilient global food system.

Hazelnuts, belonging to the birch family and originating in the Mediterranean region, are one of the world’s primary commercially cultivated nuts. Türkiye is the leading producer, contributing nearly 70% of global output, followed by Italy, China, Spain, and the United States [4]. Nutritionally, hazelnuts are a rich source of protein, healthy fatty acids, vitamins, and amino acids. Their composition is primarily oil (58–64%), protein (11–16%), and carbohydrates (15–18%) [5]. Hazelnut protein is recognized for its exceptional quality, with a biological value comparable to that of animal proteins. It provides all eight essential amino acids (30.9% to the total amino acids) as well as nine non-essential ones [6]. In addition to its nutritional benefits, hazelnut protein contributes valuable functional properties to food formulations, such as fat and water absorption, emulsification, foaming capacity, gelation, and colour enhancement [7]. Despite their high nutritional value, plant-based proteins often exhibit inferior technological functionality compared to animal-derived proteins. Key limitations in solubility, emulsification, foam formation, and gelation can restrict their utility as functional ingredients in food product development [2]. Consequently, for plant proteins like those from hazelnut meal to reach their full potential in the food industry, strategies or new technologies to enhance these functional properties are a critical area of research.

Cold plasma (CP), a non-thermal and non-hazardous processing technology, has garnered significant attention in the food industry for its ability to enhance the functional properties of food ingredients [8]. As the fourth state of matter, plasma is generated by ionizing a gas, producing a complex mixture of charged particles, ultraviolet photons, free radicals, reactive oxygen species, and reactive nitrogen species [9]. These reactive species can induce targeted physical and chemical modifications in polymers at micro- to nanoscales. Crucially, unlike high-temperature plasmas, cold plasma operates at low pressure and power, preventing local thermodynamic equilibrium [10]. This characteristic makes it an exceptionally effective technology for food applications, as it operates at low temperatures, minimizes the destruction of heat-sensitive nutrients and flavor compounds, and leaves no toxic residues [9].

The functional limitations of plant proteins, despite their nutritional merit, present a major barrier to their use in food products. Cold plasma (CP) technology emerges as a superior, green alternative to conventional modification methods for overcoming these limitations. A growing number of research indicates that CP treatment can successfully improve the techno-functional properties of various plant proteins [11,12,13,14]. For instance, dielectric barrier discharge CP treatment was shown to unfold the secondary structure of peanut protein, leading to enhanced solubility, emulsion stability, and water-holding capacity [11]. Similarly, CP treatment modified the secondary and tertiary structures of soybean protein isolate, resulting in improved emulsifying and foaming properties alongside reduced allergenicity [14]. Studies on pea protein demonstrated that CP treatment enhanced its gelling properties, enabling a concentrated solution to form a gel—a feat not achievable with the native protein [15]. Furthermore, treating sunflower seed protein with CP for varying durations increased its solubility, emulsifying activity, and emulsion stability, which were correlated with observed structural changes [16].

Overall, these studies highlight the potential of cold plasma technology to enhance the techno-functional properties of plant proteins. However, the effects of cold plasma treatment on protein functionality can differ depending on factors such as treatment duration, applied frequency, and protein type. To date, no research has specifically examined the modification of hazelnut protein using cold plasma technology. Therefore, the present study aims to investigate the influence of different atmospheric cold plasma (ACP) treatment times on the physicochemical and techno-functional properties of hazelnut proteins. Protein morphology was characterized through particle size analysis, zeta potential measurement, and scanning electron microscopy, while structural modifications were evaluated using FTIR spectroscopy. Finally, the practical functional outcomes were quantified by evaluating protein solubility, emulsifying activity, emulsion stability indices, and gel-forming behavior through rheological analyses.

## 2. Materials and Methods

### 2.1. Materials

Hazelnut flour was obtained from Trend Food Company (Istanbul, Turkiye). 1-anilinonaphthalene-8-sulphonic acid (ANS) and 5′,5-dithiobis-(2-nitrobenzoic acid) (DTNB) were purchased from Sigma-Aldrich (St. Louis, MI, USA). All other chemicals were analytical-grade and were purchased from Merck (Darmstadt, Germany).

### 2.2. Hazelnut Protein Extraction

Defatted hazelnut flour was prepared according to the method of Tatar et al. [7] for the extraction of hazelnut protein concentrate. Briefly, 250 g of hazelnut flour was mixed with n-hexane at a 1:3 (*w*/*v*) ratio and stirred for 1 h. The mixture was filtered through cheesecloth, and the residue was re-dispersed in an equivalent volume of fresh n-hexane. After stirring for an additional hour, the suspension was filtered, spread on a stainless-steel tray, and dried in an oven at 40 °C for 90 min to evaporate the residual solvent. The defatted flour was then dispersed in deionized water at a 1:10 (*w*/*v*) ratio and stirred for 2 h to ensure complete hydration. The pH of the suspension was adjusted to 10.0 with 1 N NaOH and stirred for 1 h to facilitate protein solubilization. The mixture was subsequently centrifuged at 10,000× *g* for 10 min. The supernatant was collected, adjusted to pH 4.5 (isoelectric point) with 1 N HCl to precipitate the proteins, and centrifuged again at 10,000× *g* for 10 min. The resulting pellet was collected, freeze-dried for 48 h, and the obtained protein powder was stored at 4 °C until further analysis.

### 2.3. Atmospheric Cold Plasma Treatment

**Figure 1:**
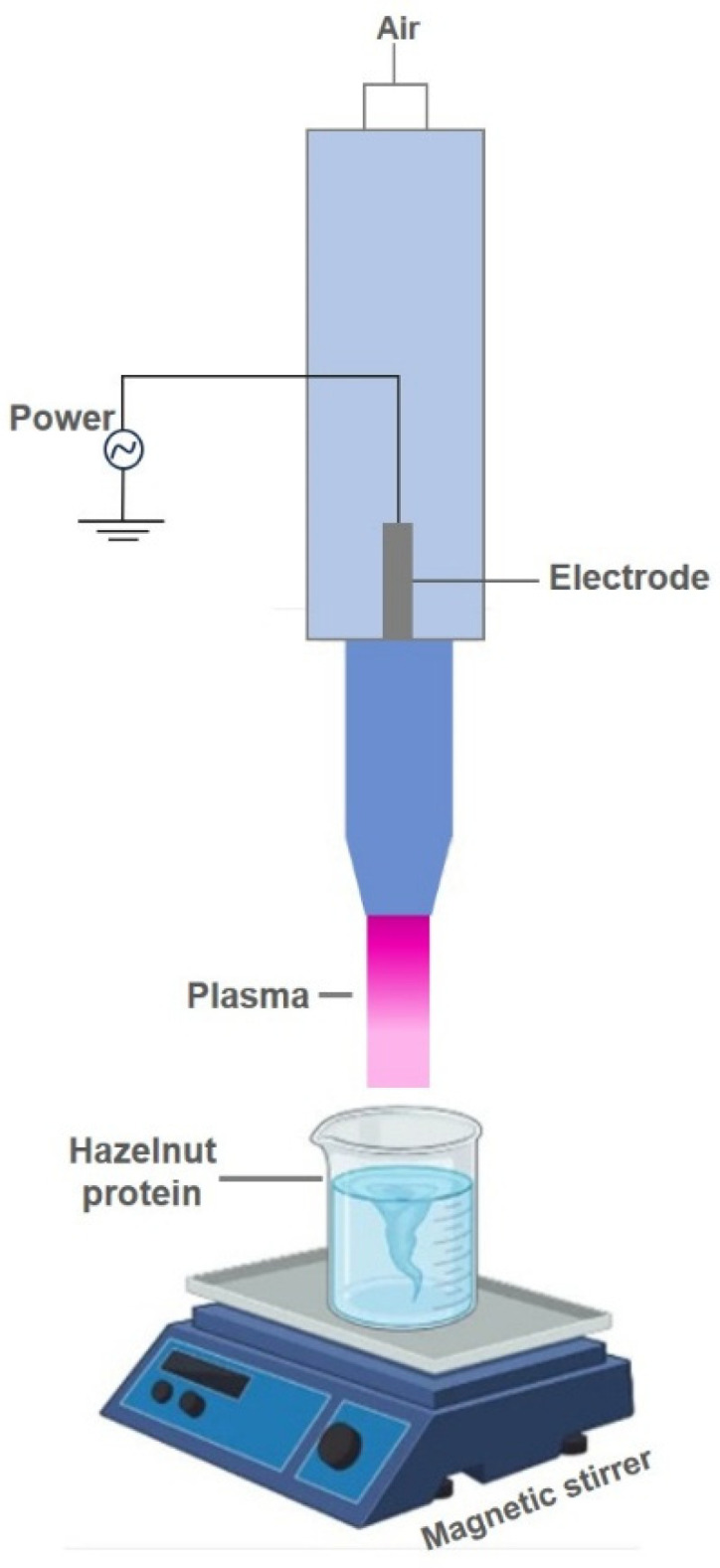
Schematic representation of atmospheric cold plasma treatment of hazelnut protein.

Atmospheric cold plasma (ACP) treatment was applied to aqueous hazelnut protein dispersions (Figure 1). The dispersions were prepared by mixing 5 g of hazelnut protein with 50 mL of deionized water and stirring continuously for 2 h prior to treatment. During ACP exposure, samples were placed on a magnetic stirrer operating at 500 rpm to maintain homogeneity, while the plasma probe was positioned 12 cm above the solution surface. Treatments were performed using an ACP system (Plasmatek A5, 600 W, Isparta, Turkiye) operating at 40 kHz, with ambient air supplied as the carrier gas at a constant flow rate of 9.42 L/min. The protein dispersions were treated for 0, 2, 4, 6, or 8 min and named as HP, HP-2, HP-4, HP-6, and HP-8, respectively. To minimize thermal effects during atmospheric cold plasma (ACP) treatment, plasma exposure was applied in 2 min intervals, allowing heat dissipation between treatment cycles. The temperature of the protein dispersions was monitored throughout the process. After ACP treatment, the final sample temperature remained within the range of 40–45 °C for all treatment durations (2–8 min). After treatment, all samples were freeze-dried for 48 h to obtain powdered proteins for subsequent analyses.

### 2.4. Characterization of Hazelnut Proteins

#### 2.4.1. Particle Size and Zeta Potential

The size and surface charge of both ACP-treated and untreated hazelnut proteins were evaluated by measuring their average particle size and zeta potential using a dynamic light scattering instrument (Zetasizer Nano ZSP, Malvern Instruments, Malvern, UK). A 1% (*w*/*v*) protein solution in distilled water was prepared and stirred for 2 h at room temperature before measurement. An aliquot (1 mL) of the prepared solution was injected into the zeta cell and the zeta potential was measured.

#### 2.4.2. Scanning Electron Microscopy

The morphology of the protein powders was analyzed using scanning electron microscopy (SEM) according to the method of Olatunde et al. [17]. Briefly, samples were directly deposited onto double-sided carbon tape adhered to aluminum SEM stubs. The stubs were then sputter-coated with a thin gold-palladium (Au/Pd) layer using a coater (Leica EM ACE 600, Heerbrugg, Switzerland) to ensure electrical conductivity. Micrographs were acquired using a field-emission scanning electron microscope (Zeiss Gemini 300, Oberkochen, Germany) at an accelerating voltage of 5 kV.

#### 2.4.3. Color Analyses

The impact of ACP treatment on hazelnut protein color properties was evaluated using a colorimeter (Konica Minolta CR-5, Tokyo, Japan). The instrument was calibrated with standard black and white reference tiles prior to analysis to ensure accuracy. Color coordinates were expressed in the color space (CIE: L*, a*, b*), with L* (lightness), a* (red-green axis), and b* (yellow-blue axis) values recorded for each sample. Total color difference (ΔE) was calculated using the following equation:

$$∆E\;=\sqrt{{\left(L\;-L*\right)}^{2}+{\left(a\;-a*\right)}^{2}+{\left(b\;-b*\right)}^{2}}$$

where L, a, and b represent the color parameters of the untreated hazelnut protein (control), and L*, a*, and b* represent the corresponding color parameters of ACP-treated hazelnut protein samples.

#### 2.4.4. Surface Hydrophobicity (H_0_)

The surface hydrophobicity (H_0_) of both untreated and ACP-treated hazelnut protein samples was assessed with ANS fluorescence probing, following an established protocol [17]. Hazelnut protein samples were first dissolved in 0.1 M phosphate buffer (pH 7.4) and centrifuged at 12,000× *g* and 4 °C for 15 min. The supernatant’s protein content was quantified via the Biuret method before serial dilution in the same buffer to achieve concentrations between 0.1 and 0.5 mg/mL. Then, 5 µL of 8.0 mM ANS solution in phosphate buffer was added to 200 µL of each diluted protein sample. After mixing and incubating in the dark for 15 min, relative fluorescence intensity was measured at 25 ± 1 °C using a Perkin Elmer FL6500 (Shelton, CT, USA) with excitation at 390 nm and emission at 470 nm (both with 20 nm bandwidths). The H_0_ value was defined as the slope of the linear regression plot of protein concentration versus fluorescence intensity.

#### 2.4.5. Free Sulfhydryl Groups

The effect of ACP treatment on the free sulfhydryl content of hazelnut protein was assessed following a modified procedure from Jin et al. [18]. In this method, 30 mg of hazelnut protein was dispersed in 3 mL of Tris–Glycine–EDTA buffer (86 mM Tris, 90 mM glycine, 4 mM EDTA, pH 8.0), vortexed for 1 min, and centrifuged at 14,000× *g* for 10 min at 4 °C. The obtained supernatant was diluted ten-fold with the same buffer before analysis. To 3 mL of the diluted sample, 30 μL of Ellman’s reagent (DTNB, 4 mg·mL^−1^ in buffer) was added, and the mixture was incubated at 25 °C for 30 min in the dark. Absorbance was recorded at 412 nm using a UV-vis spectrophotometer (SpectroStar Nano, BMG Labtech, Ortenberg, Germany). A blank sample, prepared by substituting DTNB with buffer, was included to correct for background turbidity. The free sulfhydryl content was calculated according to the following equation:

$$C_{SH}\left(\frac{\mu mol}{g}\right)=\;\frac{73.53\;\times \;A\;\times \;D}{C}$$

where *D* is the dilution factor (1.01), *C* is the diluted protein concentration determined by the biuret method (mg/mL), *A* is the absorbance measured at 412 nm.

#### 2.4.6. Fourier Transform Infrared Spectroscopy (FTIR)

Fourier-transform infrared (FTIR) spectroscopy was employed to analyze the molecular structure of hazelnut protein using a Shimadzu IRSpirit FTIR spectrophotometer (Kyoto, Japan) [19]. Spectral data were collected across the mid-infrared range (4000–400 cm^−1^) to capture vibrations corresponding to key molecular bonds, such as amide I and II bands for protein secondary structure analysis. Spectral data were acquired at a resolution of 4 cm^−1^, with 64 scans averaged per sample to enhance the signal-to-noise ratio.

#### 2.4.7. Solubility

The solubility of the hazelnut protein isolates was determined with slight modifications [2]. A 1% (*w*/*v*) protein dispersion was prepared and centrifuged at 8000× *g* for 10 min. The protein concentration in the supernatant was quantified using the biuret method. For the assay, 2 mL of the supernatant was mixed with 2 mL of biuret reagent and incubated in the dark for 30 min. The absorbance was then measured at 540 nm against a blank reagent. A standard curve prepared with bovine serum albumin (BSA) was used to determine the soluble protein concentration. The protein solubility was expressed as the percentage ratio of the soluble protein concentration in the supernatant to the total protein concentration in the initial dispersion.

#### 2.4.8. Emulsifying Activity and Stability Index

The emulsifying activity index (EAI) and emulsifying stability index (ESI) were determined according to the method of Baskinci and Gul [20]. Briefly, an emulsion was prepared by homogenizing 5 mL of sunflower oil with 15 mL of a 1% (*w*/*v*) hazelnut protein dispersion (pH 7.0) at 13,000 rpm for 2 min using a high-speed homogenizer, the Ultra Turrax T-25 (IKA Instruments, Staufen, Germany). Immediately after homogenization (t = 0) and again after 10 min (t = 10), a 30 μL aliquot was extracted from the bottom of the container and diluted in 3 mL of a 0.1% sodium dodecyl sulfate (SDS) solution. The absorbance of each diluted sample was measured at 500 nm using a SpectroStar Nano spectrophotometer (BMG Labtech, Germany). The absorbance values at t = 0 (A_0_) and t = 10 min (A_10_) were subsequently used to calculate the EAI and ESI, respectively, using the equations provided below.

$$EAI\;\left(\frac{m^{2}}{g}\right)=\frac{2\times 2.303\times A_{0}\times DF}{0.25\times C\times 10000}$$

$$ESI\;\left(min\right)=\frac{A_{0}\times 10}{A_{0}-A_{10}}$$

where dilution factor (DF) = 100, the oil volume fraction of the emulsion is 0.25, C is the concentration of the sample (mg/mL), A_0_ and A_10_ the absorbance of the diluted emulsions at 0 and 10 min, respectively.

#### 2.4.9. Foaming Capacity and Stability

The foaming capacity (FCA) and foaming stability (FST) of hazelnut protein were determined according to the method of Baskinci and Gul [20]. Briefly, 200 mg hazelnut protein (ACP treated or untreated) was dissolved in 20 mL of deionized water and homogenized at 13,000 rpm for 2 min using a homogenizer the Ultra Turrax T-25 (IKA Instruments, Germany). The foaming capacity (FC) and foaming stability (FS) were calculated based on the initial protein solution volume (V = 20 mL), and the foam volumes measured immediately after homogenization (V_0_) and after 30 min of quiescence (V_30_). The calculations were performed using the following equations:

$$FC\;\left(\%\right)\;=\frac{V_{0}\;-\;V}{V}\;\times \;100$$

$$FS\;\left(\%\right)=\frac{V_{0}-V}{V_{30}-V}\times 100$$

#### 2.4.10. Rheological Properties

Hazelnut protein gels were prepared for rheological characterization by suspending freeze-dried protein in deionized water at a concentration of 15% (*w*/*w*) and allowing the dispersion to hydrate for 4 h. The protein suspensions were then heated in a water bath at 90 °C for 30 min to induce gelation. After heating, the gels were cooled and stored at 4 °C overnight to ensure complete network stabilization prior to rheological analysis.

Dynamic rheological measurements were performed to evaluate the viscoelastic properties of hazelnut protein gels using a dynamic shear rheometer (DHR-3, TA Instruments Ltd., Crawley, UK) equipped with a 40 mm diameter parallel-plate geometry with a 1 mm gap. All measurements were conducted at 25 °C.

Strain (amplitude) sweep tests were carried out at a constant frequency of 1 Hz, with the strain varying from 0.01% to 100%, to determine the linear viscoelastic region (LVR) and assess gel resistance to deformation. Based on the LVR, frequency sweep tests were subsequently performed at a fixed strain of 0.1%, while the angular frequency was varied from 1 to 100 rad/s, and the storage modulus (G′) and loss modulus (G″) were recorded. Flow behavior was evaluated by measuring the apparent shear viscosity as a function of shear rate, which was increased from 0.1 to 100 s^−1^ at 25 °C.

### 2.5. Statistical Analysis

All experiments were performed in triplicate, and data are presented as mean ± standard deviation (SD). Statistical significance was determined by one-way analysis of variance (ANOVA) followed by Tukey’s honestly significant difference (HSD) post hoc test using SPSS software (version 16.0, SPSS Inc., Chicago, IL, USA). Differences were considered statistically significant at *p* < 0.05.

## 3. Results and Discussion

### 3.1. Particle Size and Zeta Potential

**Figure 2:**
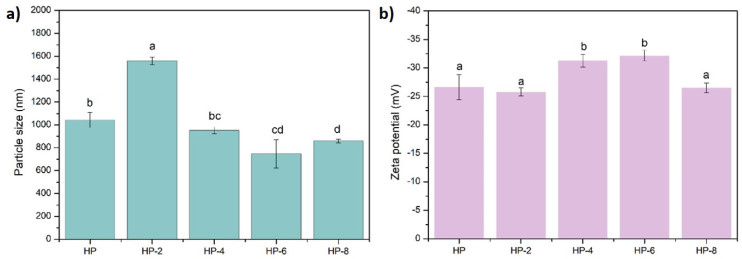
(**a**) Particle size and (**b**) Zeta potential of ACP-treated and untreated hazelnut proteins. (Different letters (a–d) represent significant differences, *p* < 0.05).

The mean particle size of hazelnut protein isolates, expressed as the hydrodynamic diameter of mesoscopic protein aggregates measured by DLS, is presented in Figure 2. The particle size ranged from 746.77 to 1560.67 nm, confirming the presence of supramolecular protein aggregates rather than individual protein molecules. ACP treatment significantly altered the particle size (*p* < 0.05). Specifically, a significant increase was observed after a 2 min treatment (1560.67 nm). However, extending the treatment duration to 4, 6, and 8 min resulted in a subsequent decrease in particle size to 952.17 nm, 746.77 nm, and 860.53 nm, respectively. This trend can be attributed to the competing effects of protein aggregation and dissociation. Initially, ACP likely induced aggregation through intermolecular cross-linking, increasing particle size. With prolonged exposure, the treatment may have decomposed these large aggregates into smaller particles, leading to a net decrease in size [21,22]. This behavior can be explained by intensified exposure to reactive oxygen and nitrogen species generated during ACP treatment, which promotes oxidative modification and cleavage of protein structures, increasing surface reactivity. While short ACP exposure (2 min) promotes partial unfolding and associative aggregation, extended treatment provides sufficient reactive species density and energy to disrupt molecular interactions and break down aggregates formed at earlier stages [23]. Consequently, smaller protein particles are generated despite increased β-sheet formation, which likely reflects intramolecular structural rearrangements within compact protein aggregates rather than intermolecular aggregation. For instance, a fivefold increase in the average particle size of buckwheat protein isolates was observed after sonication [18], while high-pressure homogenization at 150 MPa was found to reduce hazelnut protein particle size due to the breakdown of the macromolecular system [2]. Similarly, Ji et al. [11] observed a decrease in pea protein particle size up to 3 min of cold plasma treatment, followed by an increase at 4 min. Our results are consistent with the findings of Dong et al. [12], who reported a decrease in zein particle size with ACP treatment up to 3 min, attributed to the disruption of intermolecular forces between zein micelles. These findings suggest that the effect of ACP on protein particle size is a complex balance between induced aggregation and dissociation, highly dependent on treatment duration.

The zeta potential of the hazelnut protein isolates, both before and after atmospheric cold plasma (ACP) treatment, was determined to assess changes in surface charge and colloidal stability of protein aggregates (Figure 2). All samples possessed a negative charge, with zeta potential values ranging from −25.8 mV to −32.17 mV. ACP treatment significantly altered the surface charge of protein aggregates, leading to a notable increase (*p* < 0.05) in the absolute zeta potential value for proteins treated for 4 min (−31.30 mV) and 6 min (−32.17 mV). In contrast, an 8 min treatment resulted in a decrease in the absolute value (−26.5 mV). This pattern suggests that moderate ACP treatment enhances protein stability, a finding consistent with the observed reduction in particle size at these durations. The initial increase in absolute zeta potential indicates stronger electrostatic repulsion between particles, which helps prevent aggregation and improves colloidal stability. This trend aligns with studies on ACP-treated coconut globulins, where increased voltage and treatment time similarly reduced particle size and increased absolute zeta potential, until over-treatment at high voltage induced structural unfolding, hydrophobic group exposure, and aggregation [24]. The enhancement in negative surface charge is likely due to the oxidation of neutral amino acids (e.g., cysteine, methionine, and proline) into more negatively charged species during plasma treatment [25]. Conversely, the decline following extended (HP-8) treatment may result from protein surface saturation, overcrowding of micelles, and a reduction in available reactive sites, ultimately diminishing the net surface charge.

### 3.2. Scanning Electron Microscopy

**Figure 3:**
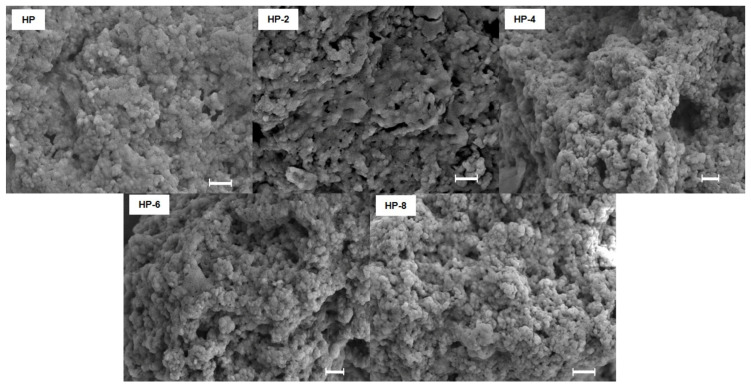
Scanning electron microscope images of hazelnut protein modified with ACP at different treatment times (Scale bar = 1 µm).

The surface morphology of hazelnut protein isolates, as visualized by scanning electron microscopy (SEM), was significantly altered by atmospheric cold plasma (ACP) treatment duration (Figure 3). Untreated proteins exhibited large, irregular aggregates with undefined shape. A 2 min ACP treatment (HP-2) resulted in an even more aggregated structure, a finding consistent with the maximum particle size recorded at this duration. However, with longer ACP treatment times (4, 6, and 8 min), the protein morphology transitioned to smaller, more distinct, and solid particles with visibly reduced aggregation. This progressive de-aggregation correlates with the increased absolute zeta potential observed at these longer durations, suggesting that enhanced electrostatic repulsion between charged protein molecules prevented flocculation and led to the formation of a more stable, dispersed system. This phenomenon is supported by previous studies on food proteins. For instance, ACP-induced oxidative modification mediated by reactive species has been shown to disrupt protein aggregates and alter surface morphology, as reported for walnut protein isolate [25]. Furthermore, the reduction of aggregates in milk protein following intermediate-duration ACP treatments has been attributed to similar surface interactions with active plasma species [26]. The underlying mechanisms are primarily attributed to ROS/RNS-mediated chemical modifications, including oxidative cleavage and structural unfolding, which alter the protein surface, reduce self-association, and improve dispersion in aqueous systems [27].

### 3.3. Color Properties

Color is a critical quality parameter influencing the consumer acceptability of proteins in food applications [23]. The impact of atmospheric cold plasma (ACP) treatment on the color of hazelnut protein isolates was evaluated by measuring the L*, a*, and b* values (Table 1). ACP treatment significantly altered all color parameters. The initial color values (L* = 52.01, a* = 11.03, b* = 25.56) were markedly influenced by the treatment duration. A significant decrease in the L* value (lightness, *p* ˂ 0.05) was observed with increasing treatment time, indicating the samples became progressively darker. The a* value (redness/greenness) exhibited an irregular trend but showed an overall decrease, culminating in a significant reduction to 10.83 after the 8 min treatment. Similarly, the b* value (yellowness/blueness) consistently decreased with extended processing, indicating a loss of yellowness. These findings align with previous studies on ACP-treated proteins, where similar non-linear trends in a-values have been reported; short durations can cause an initial increase, while prolonged treatment leads to a decrease [26,28]. ACP treatment significantly altered all color parameters, resulting in a visible darkening (decreased L*), a general decrease in redness (a*), and a loss of yellowness (b*-value). The hypothesis of Maillard reaction or lipid peroxidation can be excluded due to the negligible fat content in the samples (due to the defatting process). The total color difference (ΔE) increased with increasing ACP treatment time, with the highest ΔE (6.62 ± 1.40) observed for HP-6, indicating that prolonged ACP exposure had a greater impact on the color of hazelnut protein. Therefore, the observed color changes are solely attributed to the direct reaction between reactive plasma species and the hazelnut proteins. It is suggested that ozone and other reactive oxygen species (ROS) generated during ACP treatment, such as •OH, HO_2_•, •O_2_^−^, and •O_3_^−^, diffuse into the protein matrix and act on the aromatic rings of amino acid residues such as tyrosine, tryptophan, and phenylalanine [29]. The oxidation of these aromatic side chains and the potential formation of colored compounds like melanoidins through oxidative pathways are the most plausible mechanisms for the systematic darkening and reduction in yellowness observed in our study. The irregular trends in the a^*^-value may reflect complex, time-dependent competition between the formation and breakdown of these chromophores during extended treatment.

### 3.4. Surface Hydrophobicity (H_0_)

**Figure 4:**
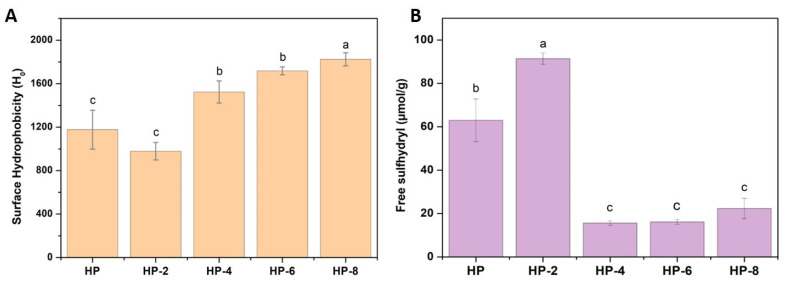
(**A**) Surface hydrophobicity and (**B**) free sulfhydryl content of hazelnut proteins. (Different letters (a–c) represent significant differences, *p* < 0.05).

Figure 4 illustrates the surface hydrophobicity and free sulfhydryl (SH) group content of hazelnut protein isolates following atmospheric cold plasma (ACP) treatment for various durations. Surface hydrophobicity serves as a critical indicator of protein functionality, quantifying the abundance of exposed non-polar amino acids available to engage in hydrophobic interactions at interfaces, such as those in emulsions and foams [30]. The surface hydrophobicity of hazelnut changed from 978.5 to 1824.5 depending on the applied ACP time. Although a slight decrease in surface hydrophobicity was observed in HP-2 protein samples, ACP-treated hazelnut proteins overall showed a rapid increase compared to the native samples (*p* ˂ 0.05), suggesting that ACP induced partial dissociation of protein aggregates and subunits, thereby altering their spatial structure. This structural rearrangement exposed previously buried hydrophobic groups, resulting in greater surface hydrophobicity [31]. Consistent with previous literature, atmospheric cold plasma (ACP) treatment is an effective method for disaggregating protein complexes and inducing partial unfolding, thereby exposing hydrophobic regions that are typically buried within the native structure [13,29,30,32]. Similar findings have been reported by Wang et al. [33], who observed a marked increase in surface hydrophobicity of chickpea protein isolates following 0–50 s of atmospheric pressure plasma, and by Chen et al. [24], who demonstrated that ACP treatment gradually enhanced the exposure of hydrophobic groups in coconut globulin at 50–60 kV. Such increases in hydrophobicity are functionally significant, as they enable protein molecules to adsorb more effectively onto oil droplet surfaces, thereby improving emulsifying capacity.

### 3.5. Free Sulfhydryl Groups

Figure 4B presents the changes in free sulfhydryl (SH) group content of hazelnut protein following atmospheric cold plasma (ACP) treatment for different durations. The free SH content increased significantly (*p* < 0.05) from 63.01 μmol/g in the untreated sample to 91.41 μmol/g after 2 min of treatment, indicating notable structural modifications in the protein. This initial increase suggests that short-term ACP exposure cleaves disulfide (S–S) bonds and disrupts tertiary structure, thereby releasing previously buried SH groups and increasing their accessibility. Later, longer exposure resulted in a sharp decline, with the content dropping to 15.65 μmol/g (HP-4). Extended treatment exposes these groups to reactive plasma species, particularly ozone, which oxidizes the newly liberated and existing thiols, leading to the reformation of disulfide bonds and a consequent reduction in free SH content. Protein oxidation is typically accompanied by the depletion of free sulfhydryl groups, with reversible products (such as disulfides and sulfenic acids) or irreversible products (such as sulfonic and sulfonic acids) generated depending on the oxidative conditions [14,29]. Therefore, the observed loss of sulfhydryl groups in hazelnut proteins under ACP treatment can be attributed to the oxidation of aromatic or sulfur-containing amino acid residues [29]. Similar results have been reported in other proteins treated with ACP; for instance, Zhang et al. [14] reported a reduction in free SH groups in soy protein isolate regarding ACP processing time, which led to degradation of sulfhydryl groups in sulfur-containing amino acids. Segat et al. [29] related the decrease in free sulfhydryl content of whey protein with the extent of ACP process to the loss of amino acids such as cysteine and the formation of new disulfide cross-links.

### 3.6. FTIR

**Figure 5:**
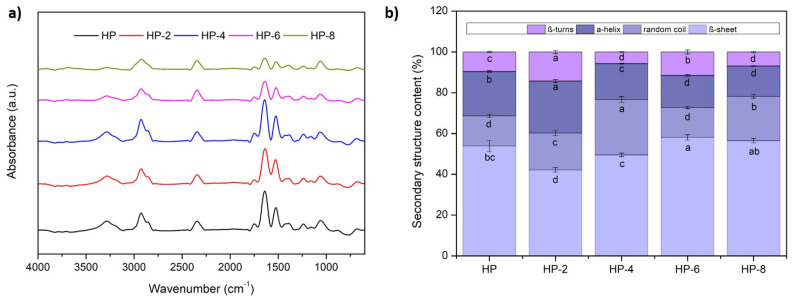
(**a**) Fourier-transform infrared (FTIR) spectra and (**b**) secondary structure content of untreated and ACP-treated hazelnut proteins. (Different letters (a–d) represent significant differences, *p* < 0.05).

Fourier-transform infrared (FTIR) spectroscopy is an effective tool for examining molecular-level interactions in composite materials. Changes in the positions of absorption peaks provide insight into alterations among functional groups and chemical bonds, enabling detailed structural analysis and interaction evaluation [34]. Notably, the absorption bands observed at 1700–1600 cm^−1^ and 1600–1500 cm^−1^ correspond to amide I and amide II, respectively, which are key indicators of protein secondary structure and molecular conformation [35]. The amide I bands are mainly attributed to the C=O stretching vibrations of the peptide backbone, while the amide II band results from N–H bending and C–N stretching vibrations [24]. The secondary structure of proteins can be sensitively probed in the 1700–1600 cm^−1^ spectral region (amide I band), where specific absorption peaks are characteristic of different conformational elements. The peaks occurring at 1650–1660 cm^−1^ are typically assigned to α-helices, those between 1610–1640 cm^−1^ and 1682–1700 cm^−1^ to β-sheets, the region from 1661–1681 cm^−1^ to β-turns, and absorptions from 1640–1650 cm^−1^ to random coils [36]. In this study, the relative percentages of these secondary structural elements in the hazelnut protein were quantitatively determined from the deconvoluted amide I band; the values presented in Figure 5b were calculated from the integrated areas of the individual peaks identified through Gaussian curve fitting.

ACP treatment significantly altered the secondary structure of hazelnut proteins. A 2 min treatment decreased β-sheet content while increasing the proportions of random coils, α-helices, and β-turns. In contrast, longer ACP treatments (>2 min) increased β-sheet content while reducing α-helix and β-turn structures, a trend consistent with previous reports [30,33]. These changes suggested that ACP induces partial unfolding and structural reorganization, facilitating the conversion of α-helices and β-turns into β-sheets [32]. The aggregation of proteins under prolonged plasma exposure is driven by a hydrophobic mechanism. An increase in β-sheet structures enhances overall protein hydrophobicity, which intensifies intermolecular hydrophobic forces. This drives aggregation and further stabilizes the β-sheet formations [33]. Moreover, the observed reduction in α-helix content suggests the disruption of hydrogen bonds between amide groups and surrounding water molecules, which is indicative of increased molecular flexibility and unfolding induced by plasma treatment. This aligns with observations in zein protein, where extended ACP treatment decreased α-helix, random coil, and β-turn content while increasing β-sheets [37]. Similarly, Wang et al. [33] noted a reduction in α-helix and random coil structures alongside increased β-sheet formation in chickpea proteins treated with an atmospheric pressure plasma jet.

The amide II region, spanning 1575–1480 cm^−1^, is associated with N–H bending vibrations and C–N stretching, often reflecting changes in hydrogen bonding and conformational states. FTIR analysis revealed that following 2 min of ACP treatment, the peak intensity at 1515 cm^−1^ increased and shifted to a lower wavenumber (1509 cm^−1^), though prolonged treatment resulted in a slight reduction in intensity. These spectral alterations suggest that amide groups within the hazelnut protein may have become increasingly involved in hydrogen bonding or were further embedded within hydrophobic regions of the protein, possibly due to structural rearrangement [38]. The observed changes are consistent with partial unfolding of the protein structure, likely induced by collisions with high-energy ions and reactive species generated during plasma treatment, which can disrupt intermolecular interactions and promote conformational changes.

Additional changes were observed in other spectral regions. The broad band at 3500–3100 cm^−1^, associated with O–H and N–H stretching vibrations (amide A), became broader and less intense following ACP treatment. Similarly, the intensities of bands at 2855 cm^−1^ and 2924 cm^−1^, corresponding to symmetric and asymmetric –CH_2_ stretching vibrations, progressively decreased with longer treatment durations [24]. These modifications indicate that ACP affects both hydrogen bonding and hydrophobic interactions: reduced N–H/O–H intensity suggests disruption of hydrogen-bonding networks and partial protein unfolding, while diminished –CH_2_ signals may reflect oxidative degradation of aliphatic side chains. Collectively, these spectral changes point to ACP-induced conformational rearrangements and chemical modifications, including oxidation, structural unfolding, and potential aggregation of hazelnut proteins.

### 3.7. Solubility

Protein solubility, a key thermodynamic property governing protein functionality, is determined by the equilibrium between protein–protein and protein-solvent interactions. This balance is influenced by internal structural factors—such as amino acid composition, molecular weight, and the distribution of hydrophilic and hydrophobic surface groups—as well as external conditions including pH, ionic strength, temperature, and solvent composition [39,40]. Moreover, solubility is intrinsically linked to protein denaturation and aggregation behavior, and it critically affects functional properties like emulsification, foaming, gelation, and sensory quality [40,41]. As many protein-based applications (e.g., emulsions, foams) require adequate solubility, their profile across pH ranges serves as a practical indicator of potential functionality [40,42].

**Figure 6:**
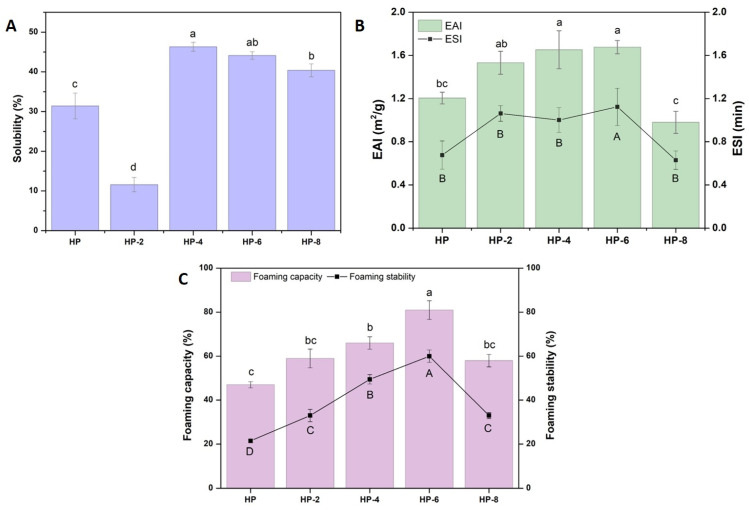
(**A**) Solubility, (**B**) emulsifying activity (EAI) and stability indices (ESI), and (**C**) foaming capacity and stability of hazelnut proteins after ACP treatment for 0, 2, 4, 6, and 8 min. Different letters (a–d and A–D) indicate a significant difference between the groups (*p* ˂ 0.05).

The effect of atmospheric cold plasma (ACP) treatment time on the solubility of hazelnut protein isolates is presented in Figure 6. ACP application significantly (*p* < 0.05) altered protein solubility, which ranged from 11.58% to 46.32%. The solubility of the untreated protein was 31.41%. A 2 min treatment (HP-2) markedly reduced solubility to 11.58%, whereas longer treatments (4–6 min) considerably enhanced it, yielding values of 46.32% (HP-4) and 44.12% (HP-6) that exceeded the untreated sample. This biphasic trend corresponds with particle size measurements: the initial decline in solubility coincides with increased aggregation at 2 min, while the subsequent improvement aligns with reduced particle size after longer treatments. The initial solubility drop is attributed to protein aggregation driven by ACP-induced structural changes. The significant increase in free sulfhydryl groups indicates cleavage of internal disulfide bonds, compromising the native protein structure and creating a more flexible state. However, this partial unfolding exposes localized reactive surface patches rather than extensive hydrophilic regions, promoting associative aggregation through hydrogen bonding and dipole–dipole interactions. The absence of a significant increase in surface charge further limits electrostatic repulsion, facilitating aggregation [43] and reducing protein–water interactions, thereby lowering solubility. In contrast, prolonged ACP exposure (≥4 min) increased solubility. Extended exposure promotes ROS/RNS-mediated oxidative modification and oxidative cleavage of protein structures, leading to the breakdown of early-stage aggregates, exposed hydrophilic residues, reduced particle size, and improved protein dispersion. Additionally, plasma-induced oxidation and surface charge redistribution enhance protein–water interactions [40], collectively contributing to the recovery and enhancement of solubility at longer treatment durations. These findings are consistent with Nyaisaba et al. [43], who reported a similar decrease and subsequent increase in solubility for ACP-treated sesame protein isolates with extended processing time.

### 3.8. Emulsifying Activity and Stability Index

Proteins stabilize emulsions primarily by adsorbing at the oil-water interface to form a protective film and by enhancing the viscosity of the continuous phase to retard droplet coalescence. In this study, the impact of atmospheric cold plasma (ACP) treatment on the emulsifying properties of hazelnut protein was evaluated by measuring the Emulsifying Activity Index (EAI) and Emulsifying Stability Index (ESI).

ACP treatment significantly improved the EAI compared to the native protein (*p* < 0.05), with values increasing from 28.21 m^2^/g to 40.23 m^2^/g following 6 min of exposure (Figure 6). Prolonged treatment (8 min), however, resulted in a marked reduction in EAI (*p* < 0.05). Protein emulsifying properties are strongly influenced by solubility, hydrophobicity, and conformational flexibility, as these factors determine adsorption kinetics and the stability of the interfacial film. The observed enhancement can be linked to the reduced particle size of hazelnut protein after ACP treatment (Figure 2), which improved interfacial properties, increased solubility (Figure 6), and facilitated better molecular dispersion in the aqueous phase. This, in turn, promoted faster diffusion to the oil–water interface and more efficient adsorption and film formation. These effects can be attributed to the interaction of plasma-generated reactive oxygen and nitrogen species (ROS/RNS) with protein molecules, which induces mild oxidation and partial unfolding, leading to the exposure of hydrophobic groups and reactive amino acid residues [33]. Such structural changes enhance protein affinity for oil droplets and increase surface activity at the interface. Accordingly, the emulsifying activity index (EAI) was closely associated with surface hydrophobicity, as proteins with higher surface hydrophobicity typically exhibit superior emulsifying performance [41]. This aligns with the increase in surface hydrophobicity observed under extended ACP processing (Figure 4), a parameter critical for interfacial behavior due to its role in protein adsorption at oil–water and oil–air interfaces.

ESI measures a protein’s ability to resist phase separation and maintain emulsion stability over time [44]. As shown in Figure 6, ACP treatment significantly enhanced this property. The ESI of the native protein (20.6 min) increased to 41.08 min following a 6 min treatment. Although prolonged treatment (8 min, HP-8) resulted in a decrease from this peak, the ESI remained significantly higher than that of the native protein. This improvement can be attributed to the ACP-induced exposure of hydrophobic groups, which promotes the formation of a more robust and cohesive interfacial film, thereby reducing interfacial tension and enhancing emulsion stability [10]. This finding is consistent with the literature, which suggests that a reduction in α-helical content can increase surface hydrophobicity, a property favorable for emulsion stability [45]. A similar non-linear trend was observed in egg white protein, where stability increased with ACP duration up to an optimum point before declining at longer treatment times.

### 3.9. Foaming Capacity and Foaming Stability

Foaming capacity (FC) and foaming stability (FS) are critical functional properties that determine a protein’s applicability in aerated food products [46]. ACP treatment significantly modulated these properties in hazelnut protein. Both FC and FS increased with treatment time up to an optimum at 6 min (*p* < 0.05), after which a significant decrease was observed at 8 min; however, the values at 8 min remained higher than those of the untreated control.

This initial improvement can be attributed to several interrelated factors. The observed increase in protein solubility (Figure 6) facilitated a faster diffusion and adsorption of protein molecules to the air–water interface [47]. Furthermore, ACP treatment is known to disrupt hydrophobic interactions and ionic bonds, increasing molecular flexibility and enhancing the ability to reduce surface tension [48]. The concomitant disintegration of large protein aggregates likely contributed to the enhanced foam stability by promoting the formation of a more cohesive and viscoelastic interfacial film. The decline in FC and FS beyond the 6 min optimum is likely due to the detrimental effects of excessive treatment. Prolonged ACP exposure may lead to an over-oxidation of proteins via high concentrations of reactive oxygen and nitrogen species [46]. This can induce excessive aggregation, resulting in large, inflexible protein complexes with reduced mobility and capacity to stabilize the air–water interface. The trends in foaming properties showed a strong positive correlation with the protein solubility profile (Figure 6), confirming the fundamental link between protein dispersibility and interfacial functionality. It is noteworthy that while ACP treatment significantly altered foaming performance (*p* ˂ 0.05), it did not affect the macroscopic heterogeneity of bubble sizes within the foams; all samples exhibited a range of large, heterogeneous bubbles.

### 3.10. Rheological Evaluation of Hazelnut Protein Gels

Rheological measurements were conducted to evaluate the viscoelastic behavior and structural robustness of gels prepared from hazelnut protein subjected to ACP treatment for different durations. Amplitude sweep and frequency sweep tests were employed to assess gel strength, resistance to deformation, and network stability.

**Figure 7:**
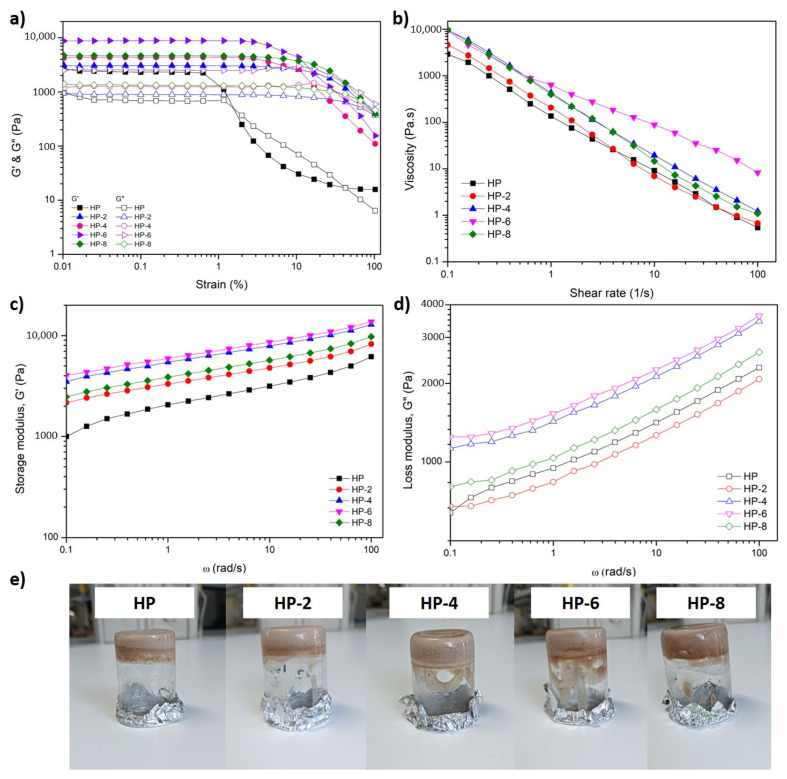
Rheological analysis of different ACP treatment durations (0, 2, 4, 6, and 8 min). (**a**) strain sweep, (**b**) viscosity profiles (flow sweep), (**c**) frequency sweep—storage modulus (G′), and (**d**) loss modulus (G″), (**e**) hazelnut protein gels.

Amplitude sweep tests (Figure 7a) were performed to determine the linear viscoelastic region (LVR) and gel resistance to deformation. The results showed that the storage modulus (G′) was consistently higher than the loss modulus (G″) for all samples at low strain levels. This behavior confirms the formation of elastic, solid-like gel networks in both untreated and ACP-treated hazelnut protein gels. The dominance of G′ over G″ within the LVR indicates that elastic interactions governed the mechanical response of the gels. As strain increased beyond the LVR, both G′ and G″ progressively decreased, reflecting the gradual disruption of the gel network. At higher strain amplitudes, G″ exceeded G′, indicating a transition from solid-like to viscous-dominated behavior as the protein network collapsed under excessive deformation. This strain-dependent breakdown is characteristic of physically crosslinked protein gels and highlights their sensitivity to large deformations.

Frequency sweep tests (Figure 7c,d) were conducted within the LVR over a frequency range of 0.1–100 rad/s and further confirmed the gel-like nature of the hazelnut protein gel systems. Across all samples, G′ values were significantly higher than G″ throughout the tested frequency range, demonstrating stable elastic behavior under small oscillatory deformations. Both moduli exhibited only slight frequency dependence, suggesting the presence of a well-developed and interconnected protein network capable of maintaining structural integrity over a wide range of deformation rates. Notably, ACP treatment had a pronounced effect on the viscoelastic properties of the gels. The G′ values increased progressively with plasma treatment time, indicating that ACP promoted the formation of stronger and more compact gel networks. Compared to the untreated control, all ACP-treated samples exhibited higher G′ values, confirming the positive role of cold plasma in enhancing gel strength. This improvement can be attributed to plasma-induced structural modifications in hazelnut proteins, such as partial unfolding, increased exposure of reactive groups, and enhanced intermolecular interactions, which collectively favor network formation during gelation.

Among the ACP-treated samples, the highest gel strength was observed for gels prepared from proteins treated for 6 min, followed by 4 min, 8 min, 2 min, and the untreated control. This non-linear response highlights that gel strengthening induced by ACP is strongly dependent on the balance between protein unfolding, intermolecular interactions, and oxidative modifications [49,50]. The enhanced G′ values at intermediate treatment times (4–6 min) can be directly associated with the pronounced increase in surface hydrophobicity observed after ACP processing. ACP treatment partially dissociated protein aggregates and induced conformational rearrangements, leading to the exposure of previously buried non-polar amino acid residues [51]. The resulting increase in surface hydrophobicity promoted stronger hydrophobic interactions between protein molecules during gelation, facilitating the formation of a denser and more cohesive three-dimensional network [15].

In addition to hydrophobic interactions, changes in sulfhydryl (SH) group content played a critical role in regulating gel structure. Short-term ACP treatment (2 min) significantly increased free SH content, indicating cleavage of disulfide bonds and partial unfolding of protein structures. However, this treatment duration coincided with reduced solubility and relatively weaker gel strength, suggesting that excessive aggregation driven by newly exposed reactive groups may have prevented the formation of an optimal gel network. In contrast, intermediate ACP treatment durations (4–6 min) promoted a controlled oxidation environment in which exposed SH groups were partially converted into new disulfide bonds. The formation of these intermolecular S–S cross-links likely contributed to network stabilization and increased elastic resistance, consistent with the elevated G′ values observed for these samples. Prolonged ACP exposure (8 min) slightly reduces gel strength due to excessive protein oxidation. Higher ACP treatment durations deplete free SH groups and form irreversible oxidation products, disrupting the protein–protein interactions and junction zones necessary for a stable network. This structural decline is confirmed by a sharp drop in SH content and aligns with findings that extended plasma exposure leads to protein degradation [16].

The low frequency dependence of both G′ and G″ observed for ACP-treated gels further confirms the formation of strong, elastic networks with high resistance to deformation, particularly for the HP-4 and HP-6 samples. Overall, these findings demonstrate that ACP treatment optimally enhances hazelnut protein gelation by synergistically modulating surface hydrophobicity, sulfhydryl chemistry, and solubility. However, exceeding the optimal treatment window leads to reduced network integrity, emphasizing the importance of precise control over plasma processing conditions.

Figure 7b illustrates the apparent viscosity of untreated and ACP-treated hazelnut protein gels as a function of shear rate. All samples exhibited a pronounced shear-thinning behavior, characterized by a continuous decrease in viscosity with increasing shear rate. This behavior is typical of protein-based gel systems and reflects the progressive disruption and alignment of the three-dimensional network structure under applied shear [52]. ACP-treated hazelnut protein gels consistently exhibited higher viscosity than the untreated control, except HP-2, confirming that plasma modification strengthens the gel network. The highest viscosity was observed for the HP-6 samples, followed by HP-4 and HP-8, consistent with trends observed in storage modulus (G′). These results indicate that moderate ACP treatment (4–6 min) promotes optimal protein unfolding and intermolecular interactions, leading to stronger and more shear-resistant gel structures [53]. On the other hand, 2 min-ACP promoted associative protein aggregation and reduced hydration efficiency, leading to a less continuous and weaker gel network and, consequently, lower viscosity in HP-2 compared with the untreated control (HP) despite the increased particle size (Figure 2a). The reduced viscosity of HP-2 gels can therefore be attributed to aggregation-dominated behavior and limited protein solubility at short treatment durations, which restricts effective network development. The slight decrease in viscosity observed for HP-8 gels suggests that prolonged plasma exposure may induce excessive structural modification or oxidation, partially weakening the gel matrix [53].

Similar plasma-induced viscosity enhancement has been reported for oat protein dispersions, where cold plasma-treated samples exhibited consistently higher viscosity than the control, with the greatest effect observed at intermediate treatment conditions, followed by samples subjected to more intense treatments [49]. Moreover, Amirabadi et al. [54] reported a five-fold increase in the apparent viscosity of gum Arabic dispersions after 20 min of cold atmospheric plasma (CAP) treatment, with the maximum effect occurring at low shear rates. The increase was attributed to strengthened intermolecular linkages, particularly hydrophobic interactions and hydrogen bonding, induced by plasma treatment. However, prolonged plasma exposure resulted in a gradual decrease in viscosity, suggesting over-modification or partial degradation of the macromolecular structure. Overall, the flow sweep results confirm that ACP treatment enhances both gel strength and resistance to shear deformation, supporting its effectiveness in tailoring the functional properties of hazelnut protein gels.

## 4. Conclusions

This study investigated the impact of atmospheric cold plasma (ACP) on the physicochemical and techno-functional properties of hazelnut protein. ACP treatment significantly reduced protein particle size and increased the absolute zeta potential, with the most pronounced effects observed after 6 and 8 min. Morphological analysis via scanning electron microscopy (SEM) confirmed protein aggregation after a 2 min treatment, followed by dissociation and size reduction at longer durations. It is proposed that partial protein unfolding during ACP exposure induced conformational changes, leading to increased surface hydrophobicity. This enhancement in hydrophobicity directly contributed to superior emulsification activity and stability, as well as foaming capacity and stability. Atmospheric cold plasma treatment enhanced gel strength and viscosity, with the strongest network observed at intermediate treatment time (6 min), due to improved protein–protein interactions and network density. Longer treatment slightly weakened the gels, indicating that excessive plasma exposure may over-modify protein structures and reduce network integrity. Overall, hazelnut protein treated with ACP for 6 min exhibited the most improved functional properties. Future studies should focus on elucidating the effects of ACP on the gelation properties of hazelnut proteins to fully assess its potential as a non-thermal modification technique.

## Figures and Tables

**Table 1:** Color properties of hazelnut proteins.

	L*	a*	b*	ΔE
HP	52.01 ± 0.96 ^a^	11.03 ± 0.06 ^bc^	25.56 ± 0.40 ^a^	-
HP-2	49.37 ± 0.57 ^b^	11.36 ± 0.09 ^a^	25.23 ± 0.15 ^ab^	2.71 ± 1.37 ^b^
HP-4	48.03 ± 1.06 ^b^	11.03 ± 0.08 ^bc^	24.56 ± 0.43 ^bc^	4.10 ± 0.84 ^ab^
HP-6	45.69 ± 0.60 ^c^	11.11 ± 0.10 ^ab^	23.59 ± 0.13 ^ab^	6.62 ± 1.40 ^a^
HP-8	47.52 ± 0.38 ^bc^	10.83 ± 0.13 ^c^	24.29 ± 0.21 ^c^	4.67 ± 0.85 ^ab^

Values in the same column followed by different letters are significantly different (*p* < 0.05).

## Data Availability

The original contributions presented in this study are included in the article. Further inquiries can be directed to the corresponding author.
